# Pathology of Seminoma Coinciding With Small Lymphocytic Lymphoma/Chronic Lymphocytic Leukemia

**DOI:** 10.7759/cureus.16509

**Published:** 2021-07-20

**Authors:** Katherine M Bui, Hailing Zhang, Bernadette M Boac, Monica S Chatwal, Jasreman Dhillon

**Affiliations:** 1 Pathology, Moffitt Cancer Center, Tampa, USA; 2 Pathology and Cell Biology, University of South Florida Morsani College of Medicine, Tampa, USA; 3 Genitourinary Oncology, Moffitt Cancer Center, Tampa, USA

**Keywords:** seminoma, pathology, diagnosis, oncology, chronic lymphocytic leukemia, small lymphocytic lymphoma

## Abstract

We present the case of a 71-year-old male with an incidental diagnosis of seminoma coinciding with small lymphocytic lymphoma/chronic lymphocytic leukemia in the retroperitoneum. This case report illustrates the cytology, histology, immunohistochemistry, flow cytometry, and fluorescence in situ hybridization features of this exceptional case and sheds light on the importance of a collaborative multidisciplinary team in delivering quality patient care.

## Introduction

The number of cancer-related deaths in the United States has declined over the last several decades; however, it remains the second leading cause of death with a projected 608,570 deaths in 2021. Non-Hodgkin lymphoma is the seventh most common cancer among men, making up approximately 5% of male cancer diagnoses and expected to account for 20,720 deaths of men and women [1]. Small lymphocytic lymphoma (SLL) is a subtype of non-Hodgkin lymphoma, and the leukemic counterpart is chronic lymphocytic leukemia (CLL). Both SLL and CLL are the same disease of small, mature B lymphocytes, but CLL predominantly involves the peripheral blood and bone marrow with a mature, monoclonal lymphocytosis equal to or greater than 5 x 10^9^/L, while SLL presents with a predominant extramedullary disease distribution and less than 5 x 10^9^/L circulating leukemic cells [2].

Germ cell tumors are most often associated as being primary to the testis, but they may arise elsewhere, including the pineal gland (germinoma), mediastinum, and retroperitoneum. Over a nearly 35-year period, there were 19,959 testicular germ cell tumors reported versus 1,211 extragonadal germ cell tumors [3]. Seminoma is the most common testicular germ cell tumor accounting for 50% of all germ cell tumors [4,5]. It commonly occurs in men aged 30-49 years and is rare in men older than 70 years of age [6].

A diagnosis of synchronous SLL/CLL and seminoma is exceptionally rare [7], with limited medical literature on this topic. We report a case of a patient with newly diagnosed, simultaneous seminoma and SLL/CLL, focusing on diagnostic features, including cytology, histology, immunohistochemistry (IHC), flow cytometry, and fluorescence in situ hybridization (FISH) studies. Here, we emphasize the importance of clinical, radiological, and pathological correlation.

## Case presentation

**Figure 1:**
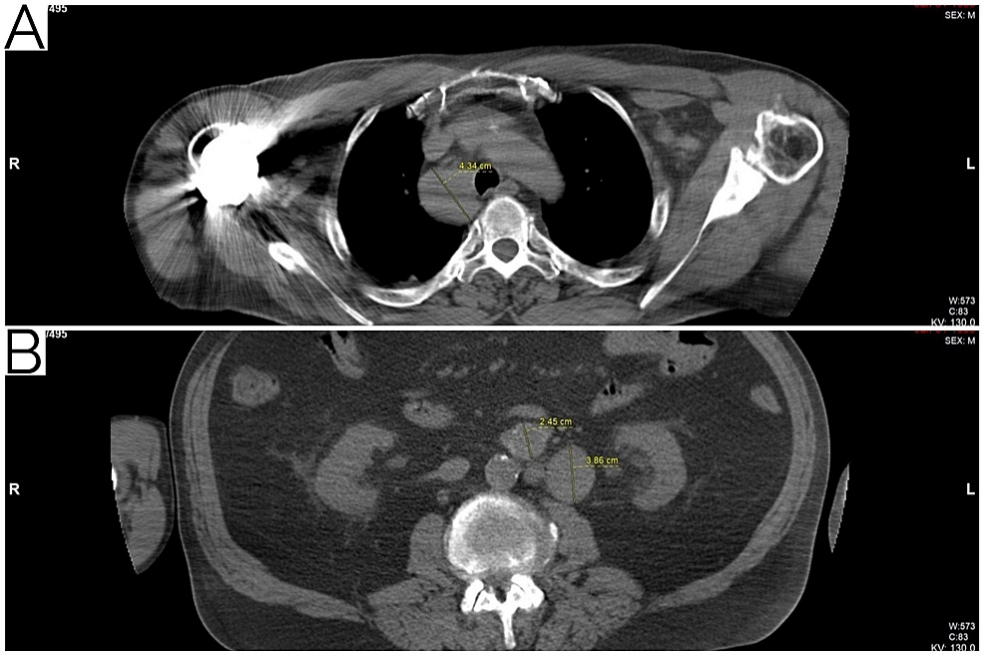
Baseline radiology. Baseline radiology of pathologic lesions, (A) right paratracheal mass and (B) left retroperitoneal mass, on CT correlates of PET imaging. CT, computed tomography; PET, positron emission tomography.

A 71-year-old male with a history of hypertension, hyperlipidemia, and asthma presented with incidentally noted retroperitoneal and paratracheal mass lesions concerning for malignancy, essentially with an unknown primary. Positron emission tomography/computed tomography (PET/CT) imaging demonstrated a metabolically active right paratracheal mass, measuring 3.9 x 4.3 cm (SUV max 6.2) and an ovoid left retroperitoneal mass, measuring 3.9 x 3.4 cm (SUV max 7.4) with several additional smaller retroperitoneal masses (Figure 1). No primary lesions were noted in the lungs, renal collecting system, bladder, or skeleton and no additional lymphadenopathy was noted. Multiple small lung granulomas were present. On initial evaluation, lymphoma was favored.

**Figure 2:**
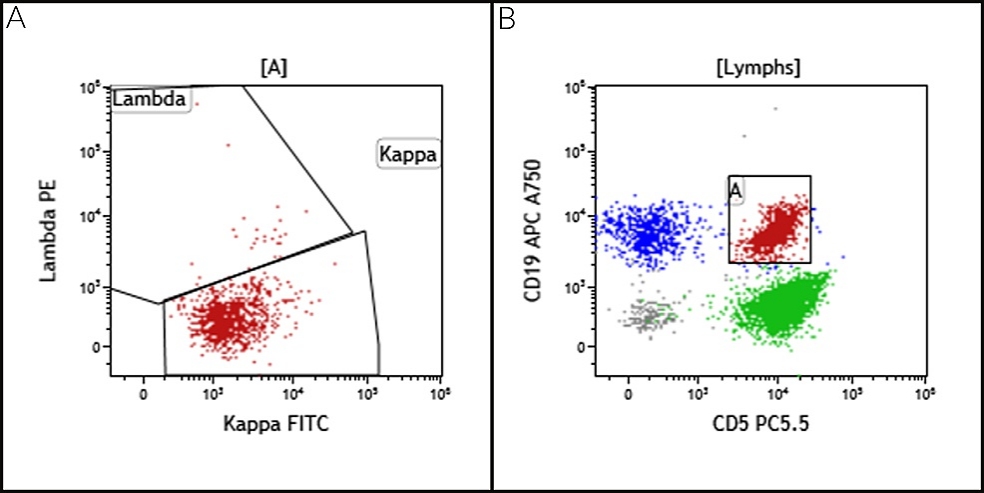
Flow cytometry. (A) Monoclonal kappa light chain-restricted B-cells detected, (B) co-expressing CD5. FITC, fluorescein isothiocyanate.

**Figure 3:**
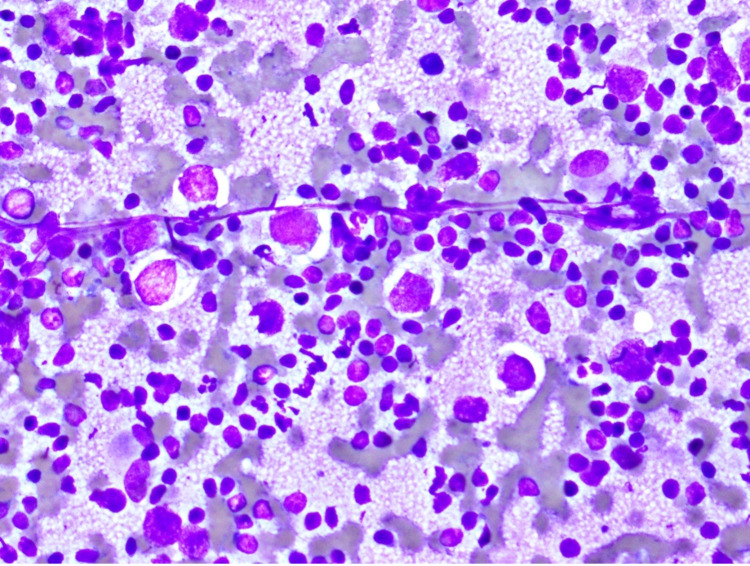
Seminoma with a background of SLL/CLL cytology. Large seminoma cells with a “tigroid” matrix and background of SLL cells (Diff-Quick stain, x400 magnification). SLL, small lymphocytic lymphoma; CLL, chronic lymphocytic leukemia.

Fine-needle aspiration and core-needle biopsies of the retroperitoneal mass were performed. The specimen was received in a total volume of 5 mL RPMI that was submitted for flow cytometry study. A monoclonal kappa light chain-restricted B-cell population was identified that was positive for CD5, CD19, CD20 (dim), CD22 (dim), CD23, and CD43 and was negative for CD3, CD10, CD38, and FMC-7, consistent with a low-grade B-cell lymphoma with a classic SLL/CLL phenotype (Figure 2). The tumor cells were negative for del(11q23), del(13q), p53/del(17p), trisomy 12, and IgH-CCND1 rearrangement by FISH study, excluding the diagnosis of mantle cell lymphoma. Cytology smears were composed of scattered, monomorphic small lymphoid cells with condensed chromatin and scant cytoplasm. Scattered within these lymphoid cells were large, atypical cells with round nuclei and prominent nucleoli with a tigroid background. These atypical cells had an abundant amount of clear-to-eosinophilic cytoplasm (Figure 3). These features were suggestive of a second malignancy that was consistent with seminoma in correlation with the concurrent core biopsy.

**Figure 4:**
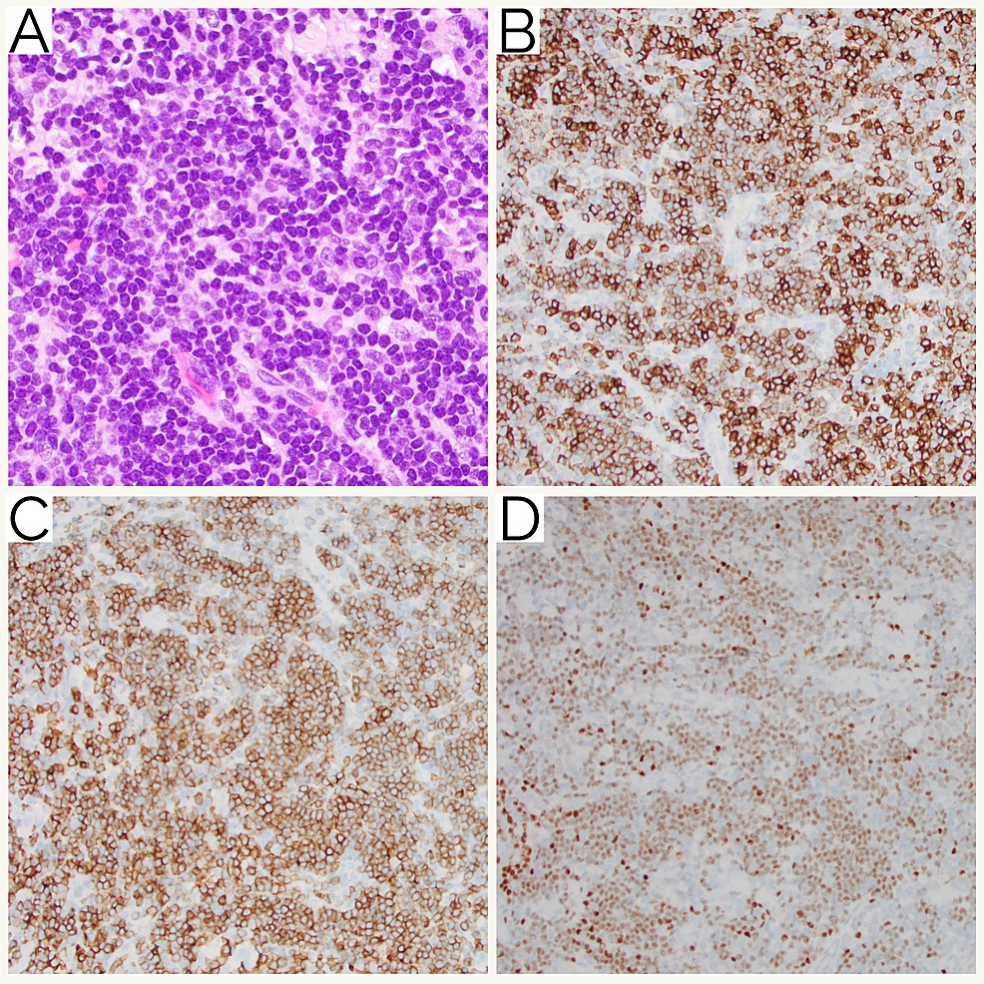
SLL/CLL histology. (A) The biopsy involved by SLL/CLL showing sheets of small lymphocytes with scant cytoplasm and clumped chromatin (hematoxylin and eosin stain, x400 magnification). Immunohistochemistry showing positive staining with (B) CD20, (C) CD5, and (D) LEF-1 (x200 magnification). SLL, small lymphocytic lymphoma; CLL, chronic lymphocytic leukemia.

Immunohistochemical studies were performed with adequate controls on the concurrent needle-core biopsies. The small lymphoid cells were predominantly composed of neoplastic CD20-positive B-cells with scattered CD3-positive T-cells. The neoplastic B-cells were also positive for CD45, PAX5, CD5, and LEF-1 and negative for cyclin D1 (Figure 4). Ki-67 proliferation index was less than 5% by manual morphometric analysis. The histology and immunostaining pattern confirmed the diagnosis of SLL/CLL.

**Figure 5:**
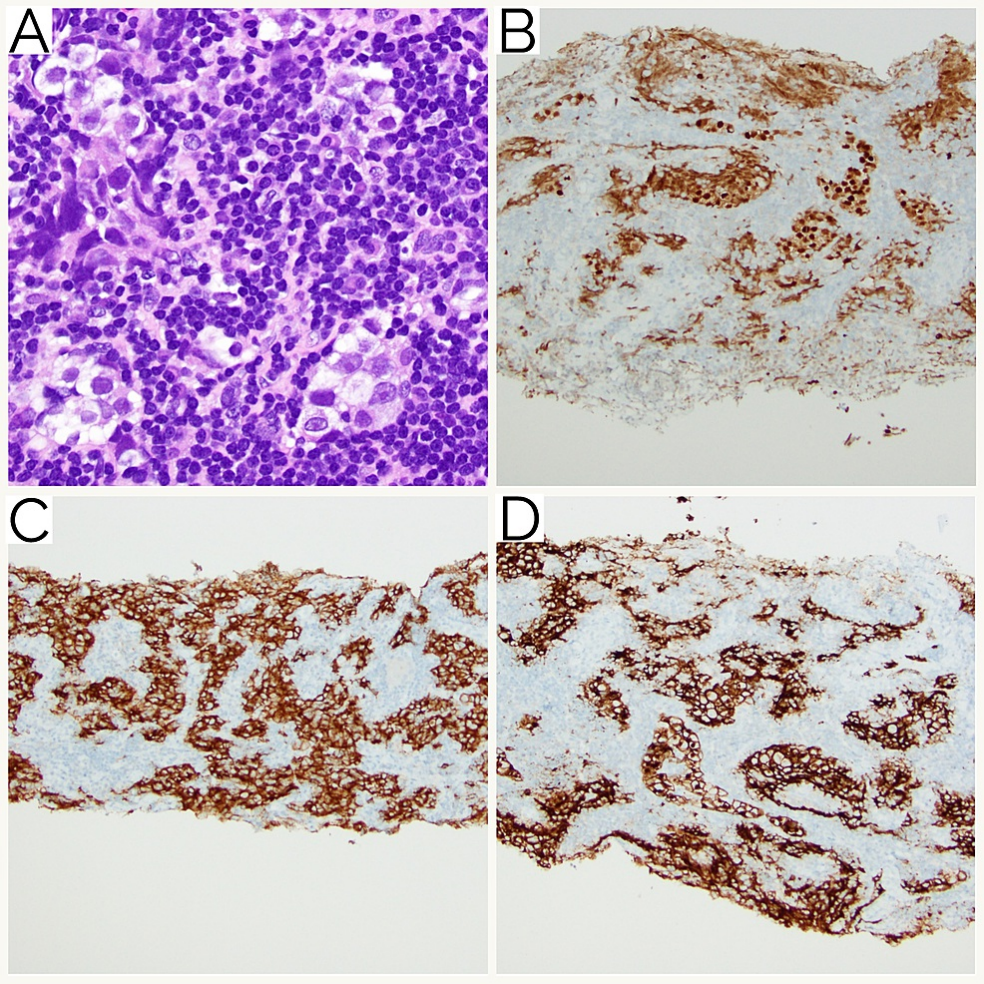
Histology of seminoma with SLL/CLL. (A) Small groups of a second population of neoplastic cells with a round nucleus, prominent nucleolus, and moderate amount of clear cytoplasm consistent with seminoma admixed with SLL/CLL (hematoxylin and eosin stain, x200 magnification). Immunohistochemistry showing positive staining of the large atypical seminoma cells with (B) OCT4, (C) CD117, and (D) PLAP (x100 magnification). SLL, small lymphocytic lymphoma; CLL, chronic lymphocytic leukemia.

Review of the sections showed clusters and nests of large, atypical cells in the background of the small mature lymphocytes. The large, atypical cells had round nuclei, prominent nucleoli with a moderate amount of clear-to-eosinophilic cytoplasm. These cells were morphologically different from the small lymphoid cells in the background. For the second population of large, atypical cells, the differential diagnosis included germ cell tumor including seminoma, sarcoma (including rhabdomyosarcoma, epithelioid leiomyosarcoma, histiocytic sarcoma, myeloid sarcoma, and epithelioid sarcoma), carcinoma including prostatic adenocarcinoma, and melanoma. The atypical large cells were positive for SALL4, OCT4, CD117, and PLAP and were negative for CD45, MDM2, CDK4, CD68, CD163, desmin, SMMS-1, myogenin, CD30, ALK1, AE1/AE3/CAM5.2, lysosome, CD138, CD33, CD43, CD34, HMB45, SOX10, NKX3.1, and SDHB. INI1 was retained. Ki-67 proliferation index was approximately 20% in this second population of cells by manual morphometric analysis. These findings confirmed the diagnosis of seminoma (Figure 5). 

**Figure 6:**
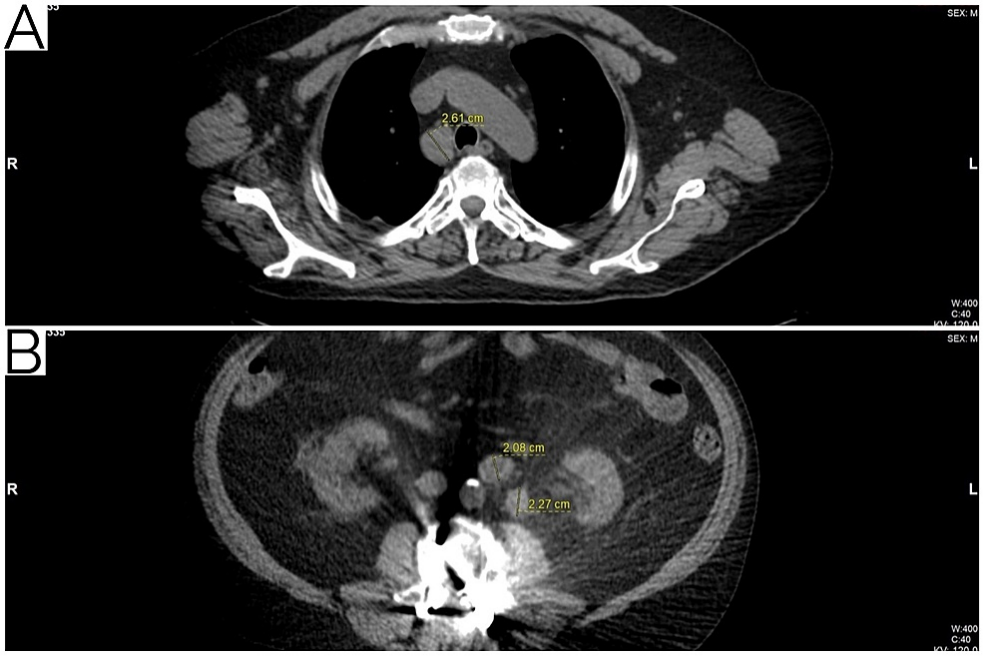
Post-treatment radiology. Post-treatment radiology of pathologic lesions, (A) right paratracheal mass and (B) left retroperitoneal mass, on CT correlates of PET. CT, computed tomography; PET, positron emission tomography.

This case was reviewed at the Moffitt Cancer Center Multidisciplinary Genitourinary Oncology and Leukemia/Lymphoma tumor boards. Interestingly, no primary testicular mass was identified in this patient by ultrasound study. Laboratory data at baseline included beta-hCG 3.67 mIU/mL (reference range, 0.00-1.90 mIU/mL), lactate dehydrogenase 188 U/L (reference range, 135-225 U/L), and alpha fetoprotein 0.8 ng/mL (reference range, 0.00-8.3 ng/mL). Complete blood count (CBC) revealed mild anemia and lymphopenia. A pulmonary function test (PFT) showed a moderate obstructive defect. Given the radiographic, pathologic, and laboratory findings, he was diagnosed with good-risk metastatic seminoma to retroperitoneal lymph nodes and right paratracheal mass (cTx N2 M1a S0, Stage IIIC) with an unspecified primary location, and SLL/CLL (Stage IIA). At that time, the decision was made to move forward with therapy. The treatment of choice was systemic therapy with primary chemotherapy for metastatic seminoma. Given the PFTs and some underlying pulmonary disease, the patient’s age, and no primary testicular mass, standard-dose etoposide and cisplatin was favored. The patient completed the planned four total cycles of chemotherapy. Follow-up imaging with PET/CT demonstrated a decrease in metabolic activity and size of the right paratracheal mass, measuring 2.1 x 2.6 cm (SUV max 2.7). There was also an interval decrease in the size and metabolic activity of the previously identified left retroperitoneal mass, measuring 1.9 x 2.4 cm (SUV max 4.4) (Figure 6). No new sites of disease were reported. Given the CBC with lack of lymphocytosis, lymphadenopathy, and B-symptoms, no therapy was indicated for the SLL/CLL and he proceeded with close monitoring.

## Discussion

SLL/CLL is a low-grade non-Hodgkin lymphoma of B-cell type involving lymph nodes, bone marrow, spleen, and peripheral blood [8]. It is the most common adult (median age between sixth and seventh decade) leukemia/lymphoma in the Western world, usually with an indolent clinical course [9]. The diagnosis of SLL/CLL includes flow cytometric identification of characteristic immunophenotype with CD19- and CD20-positive monoclonal B-cells co-expressing CD5, CD23, and CD200. Biopsy of the involved lymph nodes reveals effaced lymphoid architecture by a diffuse infiltration of small lymphocytes [2]. High-grade B-cell lymphoma transformation may occur in the context of CLL, i.e. Richter transformation [9]. Treatment is tailored based on factors including clinical progression, biomarker status, and patient’s biological fitness.

Seminoma is the most common type of testicular germ cell tumor [10]. The female counter part occurring in the ovary is dysgerminoma [11]. Seminoma typically occurs in 30- to 49-year-old males and is rare in older males (>70 years of age) [6]. It usually presents with a localized mass lesion, with less than 5% presenting as metastatic disease [12]. The histology of the tumor includes sheets of large tumor cells with clear-to-eosinophilic cytoplasm, polygonal nuclei, prominent nucleoli, and a background of mature T lymphocytic infiltrate. The tumor cells are positive for IHC stains of OCT3/4, SALL4, CD117, and PLAP. This immunoprofile helps differentiate seminoma from other germ cell tumors, as well as rule out carcinoma, sarcoma, and melanoma [13]. Cytology shows large, discohesive tumor cells with round nuclei and prominent nucleoli and a background of small mature lymphocytes. A “tigroid smear” can be appreciated in hypercellular aspirates due to leakage of the glycogen present in the cytoplasm [14].

Seminoma coinciding with SLL/CLL is exceptionally rare [7]. In this case, the seminoma occurred in a patient who is older than the typical age group. Interestingly, no primary testicular mass was identified. Additionally, in the retroperitoneal location of a senior individual, differential diagnosis of mass lesions should also include metastatic carcinoma, melanoma, and dedifferentiated liposarcoma [15].

## Conclusions

The diagnosis of a coincidental tumor is exceptionally rare. The current case report of a synchronous seminoma and SLL/CLL illustrates the clinical and pathologic features of this unusual case. Awareness of these coincidental tumors is important for diagnosis, management, and treatment. In addition, it reiterates the importance of a multidisciplinary care team to give the patient the best quality care.
